# A blackberry (*Rubus *L.) expressed sequence tag library for the development of simple sequence repeat markers

**DOI:** 10.1186/1471-2229-8-69

**Published:** 2008-06-20

**Authors:** Kim S Lewers, Chris A Saski, Brandon J Cuthbertson, David C Henry, Meg E Staton, Dorrie S Main, Anik L Dhanaraj, Lisa J Rowland, Jeff P Tomkins

**Affiliations:** 1 USDA-ARS, Beltsville Agricultural Research Center, Genetic Improvement of Fruits and Vegetables Lab, Bldg. 010A, BARC-West, 10300 Baltimore Ave., Beltsville, MD 20705-2350, USA; 2 Clemson University Genomics Institute, 51 New Cherry St., 304 Biosystems Research Complex, Clemson University, Clemson, SC 29634, USA; 3 National Institutes of Health/National Institute of Environmental Health Sciences, Laboratory of Signal Transduction, Peptide Hormone Action Group, 111 TW Alexander Drive, PO Box 12233, MD F3-04 Research Triangle Park, NC 27709-2233, USA; 4 Center for Integrated Biotechnology, Dept of Horticulture and Landscape Architecture, Washington State University, 45 Johnson Hall, Pullman, WA 99164-6414, USA; 5 Monsanto Research Centre, Biotech Product Support, 44/2A Bellary Road, NH-7, Hebbal, Bangalore 560 092, India

## Abstract

**Background:**

The recent development of novel repeat-fruiting types of blackberry (*Rubus *L.) cultivars, combined with a long history of morphological marker-assisted selection for thornlessness by blackberry breeders, has given rise to increased interest in using molecular markers to facilitate blackberry breeding. Yet no genetic maps, molecular markers, or even sequences exist specifically for cultivated blackberry. The purpose of this study is to begin development of these tools by generating and annotating the first blackberry expressed sequence tag (EST) library, designing primers from the ESTs to amplify regions containing simple sequence repeats (SSR), and testing the usefulness of a subset of the EST-SSRs with two blackberry cultivars.

**Results:**

A cDNA library of 18,432 clones was generated from expanding leaf tissue of the cultivar Merton Thornless, a progenitor of many thornless commercial cultivars. Among the most abundantly expressed of the 3,000 genes annotated were those involved with energy, cell structure, and defense. From individual sequences containing SSRs, 673 primer pairs were designed. Of a randomly chosen set of 33 primer pairs tested with two blackberry cultivars, 10 detected an average of 1.9 polymorphic PCR products.

**Conclusion:**

This rate predicts that this library may yield as many as 940 SSR primer pairs detecting 1,786 polymorphisms. This may be sufficient to generate a genetic map that can be used to associate molecular markers with phenotypic traits, making possible molecular marker-assisted breeding to compliment existing morphological marker-assisted breeding in blackberry.

## Background

The recent release of two blackberry (*Rubus *L.) cultivars with the novel trait, primocane fruiting [1], has the potential to significantly expand the blackberry industry. All other blackberry cultivars produce fruit in the summer on canes called floricanes, canes that grew the year before. Primocane-fruiting cultivars produce fruit on floricanes and then produce a smaller second crop in late summer and early fall on canes that emerged in spring and are just a few months old, thereby extending the potential fruit production period for growers, marketers, and consumers. Alternatively, canes can be mown to the ground in late fall, and the year's entire crop can be produced on canes that emerge the following spring. Because the canes escape winter injury, blackberry production of this type could expand into areas previously thought to be too cold for growing blackberries. The potential effect on the industry of expanding blackberry production both seasonally and geographically has led to a desire to develop new cultivars combining primocane fruiting with other important traits like thornlessness.

The allele conferring primocane fruiting is recessive [2], thus four copies are needed for a tetraploid, tetrasomic blackberry cultivar to produce fruit on primocanes. Expression of the trait is affected by environment and plant vigor so that progeny from cross pollinations cannot always be identified visually the first or even the second fruiting year. If thousands of progeny are to be evaluated each year, it would be helpful to eliminate undesirable genotypes as early as possible. Therefore, primocane fruiting would be an excellent candidate for marker-assisted selection. Using multiple markers around the locus, blackberry breeders could identify progeny that should be primocane fruiting or carry the trait in the heterozygous state.

Until the development of primocane-fruiting cultivars, pro-active interest in molecular marker linkage map development for cultivated blackberry has been lacking and no blackberry linkage maps have been published to date. Yet, in spite of its highly heterozygous tetraploid genome, blackberry is a good candidate for the development of marker-assisted selection. In fact, blackberry breeders already use a similar method to select seedlings with another very important trait for the industry, thornlessness. Like primocane fruiting, inheritance of thornlessness is through a recessive allele at a single locus [3]. Selection for thornlessness can be done at the seedling stage based on the absence of cotyledon marginal hairs. The two traits are thought to be very closely linked rather than the epistatic effects of alleles at a single locus, because a very small number of seedlings without cotyledonary hairs will later produce thorns (J.R. Ballington, personal communication). Thus, a sort of morphological marker-assisted-selection has been used by blackberry breeders for many years, suggesting molecular marker-assisted selection for a trait like primocane fruiting has the potential for adoption.

Linkage of a molecular marker to primocane fruiting or any other trait can be established with the use of linkage mapping strategies such as the single dose restriction fragment mapping method [4] or with software specifically designed for use with tetrasomic species, such as Tetraploid Map [5]. Due to double reduction, a well saturated blackberry map derived from multiple populations will be a map of chromosome arms rather than whole chromosomes, but useful marker linkages to important traits are still quite possible.

Both molecular linkage maps and molecular markers derived from blackberry sequences are either nonexistent or unavailable. A low percentage of simple sequence repeat (SSR) primer pairs developed from other related species such as strawberry (*Fragaria *L.) have amplified products from blackberry, fewer than a third of those tested [6]. Of the 84 SSRs derived from the *R. idaeus *subsp. *idaeus *L. (red raspberry), cultivar Glen Moy [7], no more than 26% amplify a product from either of two blackberry genotypes tested, and of the 142 Rosoideae SSR primer pairs tested in that study, only 18% detected polymorphisms between the two blackberry genotypes [8].

Lewers et al., [6] generated SSR markers from strawberry EST sequences deposited in GenBank and used them to amplify strawberry DNA. Strawberry, blackberry and raspberry belong to the same sub-family, Rosoideae. Approximately 14% of the strawberry ESTs then in GenBank contained SSRs with at least five repeats of the motif. Of the primer pairs designed from sequences with six or more repeats, 68% detected polymorphisms among tested accessions, and, of the primer pairs designed from fewer than six repeats, 43% detected polymorphisms. Therefore, it is reasonable to expect that useful SSR markers could be developed from blackberry ESTs, and that a number sufficient for genetic mapping could be expected, given that enough ESTs are generated. However, currently no blackberry sequences that might be used for molecular marker development have been deposited in GenBank. The objective of this work, therefore, was to develop a blackberry EST library and use the resulting sequences to develop SSR markers.

## Results and Discussion

### Quantification of the RNA and library

The amount of total RNA extracted from 3.5 g of blackberry leaves was 1.2 mg, and 1.8 μg of mRNA was separated from the pooled total RNA. The resulting yield of cDNA from reverse transcription of the 1.8 μg mRNA was 145 ng. The entire 145 ng was ligated with 100 ng of pDONR222 vector and transformation yielded 7.6 × 10^6 ^colony forming units. A survey of the size of the insert in 96 clones revealed an average insert size of 1.7 kbp as assessed by restriction enzyme digestion and ranged from approximately 200 bases to over 2 kb long.

### Sequence analysis, contig assembly, and homology

**Figure 1:**
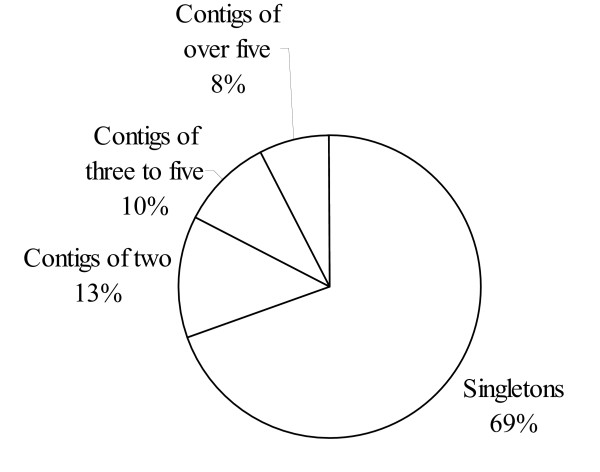
**Blackberry (*Rubus *L.) EST contig composition**. Distribution of blackberry (*Rubus *L.) EST sequences that were assembled into contigs of more than five sequences, three to five sequences, two sequences, or that remained unassembled as singletons.

After trimming, 3000 high quality sequences (77%) were obtained from the 3,884 clones selected for sequencing. The 2,678 highest quality sequences over 100 bp in length are reported as FF682838 through FF685515 in GenBank. The average read length was 889 bp with an average phred value of 28. The lower sequencing success is most likely due to the inherent formation of a secondary structure associated with the pDONR222 vector. Of these 3000 sequences, 2614 (87%) were similar (expect value < 1e-6) to sequences previously deposited in GenBank, while 386 (13%) were not. Of the 2614 sequences that were similar to others in GenBank, 580 (22% of 2614) were similar to putative proteins of unknown function. When the 3000 sequences were assembled into contigs, 919 (31%) were assembled into 301 contigs, while 2081 singletons (69%) remained separate (Fig. 1). Of the 301 contigs, 288 (96%) were similar to sequences in GenBank while 13 (4%) were not. Of the 288 contigs that were similar to other sequences in GenBank, 44 (15% of 288 contigs), containing 100 clones, were annotated as having an unknown function.

**Figure 2:**
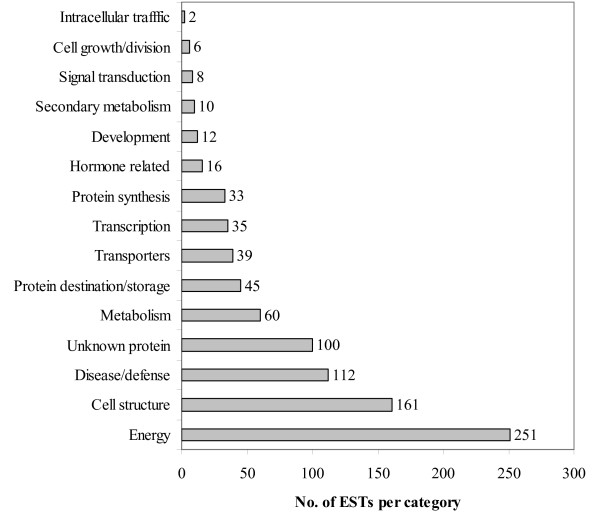
**Blackberry (*Rubus *L.) EST functional categories**. Number of blackberry (*Rubus *L.) ESTs assembled into contigs and the major functional categories into which they were classified.

The contig sequence similarities to previously reported genes were ordered by relative abundance, as determined by the number of ESTs in the contigs (Additional File 1), and were then grouped by their putative functional categories (Fig. 2) [9,10]. Among the most abundant mRNAs cloned, sequenced, and assembled into contigs were those involved with energy (especially photosynthesis and electron transport). Five contigs, consisting of 49 clones (5% of the clones assembled into contigs), had sequence similarity to the Ribulose 1,5-bisphosphate carboxylase (RUBISCO) small subunit, key to the Calvin cycle of photosynthesis. The functional category of second greatest abundance was cell structure, especially those associated with chloroplasts. Seventeen contigs, consisting of 114 clones (12% of clones in contigs), had sequence similarity to a chlorophyll a/b binding protein. These results are not surprising considering that the cDNA library was made from RNA from leaf tissue.

The third largest functional category, with 112 clones assembled into 39 contigs, was disease and defense related. Within this functional category, the majority of contigs (78 clones) were similar to genes involved with stress response, with 69 clones having similarity to heat shock proteins. Again, this is perhaps not surprising considering leaf tissue for library construction was collected from plants in July in the middle of the afternoon. Other contigs with similarity to disease and defense related genes include four (14 clones) similar to genes associated with heavy metals, four (11 clones) similar to detoxification genes, and four (9 clones) similar to disease resistance genes. The large number of clones related to disease and defense suggests that this library will also be useful for studying genes potentially associated with tolerance to abiotic and biotic stresses.

### SSR identification and testing

A total of 1,026 SSRs with 40–60% GC content and at least 20 base pairs of sequence on either side of the motif were detected and selected for primer design. Some sequences and the resulting primers were due to duplications either of the same amplification region in the sequence or two repeat regions in the same sequence. Duplications that were of the same repeat region and used the same primers were eliminated while primer pairs that amplified different regions in the same sequence and different primer pairs that amplified the same region were retained. Retention of these primer pairs was considered valuable to ensure that one working pair for each region was identified and to allow amplification of gene family members. There were 94 primer pairs of this type and 579 primer pairs that were designed from unique sequences for a total of 673 SSR containing sequences, about 22% of the 3,000 total high-quality sequences. The percentage of SSR-containing sequences is slightly higher that what was reported for strawberry (14%–15%) [6,11] and for apple (*Malus domestica *Borkh.) (17%) [12], two other Rosaceous species, and such estimates can vary depending on methods used to search ESTs for SSRs [13].

Assuming that the presence of an SSR region does not affect relative abundance of the individual mRNA in the pool, then the 673 SSR-containing sequences can be expected to be assembled into contigs in the same proportion and manner as did the total 3000 high-quality sequences obtained. Therefore, about 31% (209 sequences) are expected to be members of 68 contigs, while 69% (464 sequences) are expected to remain separate. Assuming that the sequences assembled into contigs are indeed part of the same gene and locus we can expect 68 plus 464, a total of 532 primer pairs, to amplify products from unique genomic regions.

To test the efficacy of the primer pairs, 33 were chosen randomly from the larger subset of 673 primer pairs. Sixteen of the sequences were part of nine contigs formed when the entire 3,000 sequences were considered. Considering only the subset of 33 sequences, eleven sequences formed three contigs (Additional File 2), fitting expectations based on the entire library. Of the 33 SSR primer pairs tested with 'APF-12' and 'Arapaho' DNA, 21 (64%) amplified a product, a somewhat low rate [13]. Of these 21, nine detected at least one size polymorphism, and one primer pair amplified a product from one but not the other genotype, for a total of 10 primer pairs detecting polymorphisms, around 30% of the randomly selected 33.

For one of the three contigs, Contig 219, with sequence similarity to a chlorophyll a/b-binding precursor, only half the primer pairs amplified products and did not detect polymorphisms. The other two contigs, Contigs 112 and 131, contained two sequences each, and primer pairs from both sequences in each contig amplified products, but only one primer pair per contig detected polymorphisms. Therefore, of the ten primer pairs that detected polymorphism, no two were from sequences in the same contig. These findings support the decision to test primer pairs that may amplify the same region in order to try to ensure that at least one primer pair will be useful in mapping.

The ten primer pairs amplified up to six products per genotype. The expected number of alleles per locus in a tetras genome is four, so in comparing two tetraploid genotypes with SSR primer pairs, a maximum of eight polymorphic products might be possible. Even more may be possible for loci involving genes that are duplicated in tandem repeats or loose clusters [14]. The observed number of amplification products may be lower than the expected number due to several factors, including identical product size from one or more loci and deletion of some loci as has been observed in selfed progeny of synthetic tetraploid plants [15].

The number of primer pairs detecting polymorphisms, 10 of 33 or around 30% is lower than reviews reporting ranges of 80% to 90% [16]. Most studies test new primer pairs on several different accessions of the same species, and higher levels of polymorphism would be expected, but the polymorphism rate between two individuals is far more valuable to our goal of determining usefulness of this library in genetic mapping. In addition, much of the polymorphism useful in genetic mapping is unseen in parental screens of tetrasomic blackberry. The ten primer pairs that detected polymorphisms amplified up to three polymorphic products each (present in one genotype but not the other), with an average of 1.9 polymorphisms per primer pair. When these primer pairs are used with a mapping population, the number may increase. If the locus (A for example) from which a monomorphic PCR product is amplified is present in both parents in either a singe dose (simplex = Aaaa) or a double dose (duplex = AAaa), some of the resulting segregants will be lacking the locus (nuliplex = aaaa), and some of these loci can be mapped with a software program such as TetraploidMap [5]. If there is no preferential pairing between homeologous pairs of chromosomes, segregants at a locus for which both parents have a single dose will segregate in a ratio of approximately 3:1. Segregants at a locus for which both parents have a double dose will segregate in a ratio of 35:1, and segregants at a locus for which one parent has a single dose and the other a double dose will segregate in a ratio of 11:1, even though the primer pairs amplify a product from both parents and therefore do not appear to detect a polymorphism. If the amplification products are mapped as dominant markers, it is recommended that products segregating in either 11:1 or 35:1 ratios are eliminated from the mapping data set due to difficulties in gaining information from recombination events, but that products segregating in a 3:1 ratio (product amplified from both parents) should still be included [5].

### Expectations of results from further sequencing

The fact that 69% of 3,000 sequences obtained from this library are singletons (a redundancy rate, or chance that a new sequence will already be represented in the data set, of 31%) indicates that many additional new blackberry sequences could be obtained from continued analysis of this library of 18,432 clones, even though libraries sequenced from the 5' end tend to have higher rates of insufficient overlap to form contigs, inflating the number of singletons somewhat [17]. The initial sample size is very small compared to the expected number of unique genes in any plant (even restricted to a single tissue or stage). Plants with relatively certain gene estimates, such as *Arabidopsis thaliana *(L.) Hehnh, have many more genes, and a non-normalized library can be expected to have sequence similarities to numerous genes other than the ones found in this original small sampling. It's likely that, as more sequences are obtained from the library, a greater percentage will be assembled into contigs [18,19]. Comparison of percentage singletons among other Rosaceous crops is consistent with this assumption. The percentage singletons observed in a strawberry EST library of 1,800 sequences was 65% [11], somewhat similar to what was observed in the blackberry EST library. Yet 35% of 9,984 peach (*Prunus persica *(L.) Batsch.) ESTs were singletons [20], while only 17% percent of 151,687 apple ESTs were singletons [12].

Assembly into contigs appears to help with the ability to find sequence similarity with other genes, as almost 13% of the 3,000 high quality sequences were not found to be similar to other genes in GenBank, while only around 4% of the 301 contigs formed from the 3,000 sequences had no significant similarity to other sequences in GenBank. Contigs made up of multiple ESTs have longer consensus sequences and fewer errors. This increase in both length and quality increases the probability of identifying a statistically similar protein sequence. Also, more heavily expressed genes will tend to form contigs in small samples, and higher expression makes them more likely to have been previously identified and present in databases.

## Conclusion

In summary, this paper represents the first published analysis of ESTs from cultivated blackberry. The sequences and annotation are available via GenBank and the Genome Database for Rosaceae [21]. The resulting sequences were used to develop SSR markers for eventual use in genetic mapping. A subset of 33 SSR primer pairs was predictive that the total identified 673 SSR primer pairs would detect polymorphisms at a rate of 30%, resulting in 202 SSRs detecting polymorphisms, and that these would detects an average of 1.9 polymorphisms per primer pair, or around 384 polymorphisms (Table 1). Although this number may be sufficient for identifying at least one SSR marker linked to primocane fruiting, it is unlikely to be sufficient to generate an adequate map of tetraploid blackberry. If our subset of 3, 884 clones sequenced is representative of the entire 18,432 clones picked, then we can expect 940 SSR primer pairs (22% of the sequences) from this library to detect 1,786 polymorphisms. The actual number of primer pairs that will detect polymorphisms will be lower due to redundancy which is expected to increase as more of the library is sequenced. On the other hand, the actual number of loci that can be mapped will be greater than the estimate because some of the SSR primer pairs are detecting loci present in single or double dose in both parents. The result should be a number of segregating loci sufficient to generate a fairly well saturated map that can be used by others to more efficiently identify SSR markers associated with other horticulturally important traits in other populations.

**Table 1:** Expectations of results from further sequencing of a blackberry (*Rubus *L.) EST library.

Subset sequenced	Multiplication rate	Whole library	
3884		18,432	Number of clones
	77%		
3000		14,237	Number of high quality sequences
	22%		
673		3,132	Number of SSR primer pairs to test in parental screens
	30%		
202		940	Number of primer pairs detecting polymorphisms between two genotypes
	× 1.9		
384		1,786	Number of polymorphisms to try to map

Expectations are based on results from this study of the percentage of high quality sequences obtained, the percentage of those sequences which contained SSRs, the percentage of primer pairs selected to test, the percentage of SSRs that detected polymorphisms, and the average number of polymorphic products generated from each.

## Methods

### Genotype selection and mRNA isolation

The tetraploid blackberry cultivar, Merton Thornless (PI 553276), was selected for EST development, because it is the source of the thornless trait [3] in many commercially grown thornless cultivars. Young expanding leaves of 'Merton Thornless' were harvested in July, 2004, from plants growing on the North Farm of the Beltsville Agricultural Research Center. Leaves were wrapped in aluminum foil and placed in liquid nitrogen to transport to the lab for extraction. Total RNA was extracted from 3.5 g of leaf tissue using the RNeasy Plant Mini Kit (Qiagen, Inc., Valencia, Calif.) with modifications for woody plant tissue [9]. The resulting RNA from seven extractions was pooled to increase total yield. No enrichment was done. The Poly(A)Purist™ Kit (Ambion, Austin, Tex.) was used to separate mRNA from total RNA.

### Library production and sequencing

The cDNA library was cloned in the Gateway system (Invitrogen Corp., Carlsbad, Calif.) with the pDONR222 vector and ElectroMAX™ DH10B™ T1 Phage Resistant Cells (Invitrogen Corp.) following manufacturer's instructions. Kanamycin was used to select colonies containing vectors. A total of 18,432 clones were picked and arrayed into 96-well plates for sequencing. The M13F primer and the ABI PRISM^® ^BigDye™ Terminator v3.1 reaction mix (Applied Biosystems, Foster City, Calif.) were used to sequence from the 5' end of 3884 clones. Cycle sequencing was carried out as follows: 96°C for 5 min, and 35 cycles of (96°C for 45 s, 50°C for 5 s, and 60°C for 4 min). Sequence data of 3,884 clones was accumulated from an ABI 3730xl Genetic Analyzer (Applied Biosystems).

### Trace file processing

Sequence trace files were converted into FASTA files and quality score files using the phred [22] base-calling program. Vector and host contamination (such as species specific mitochondrial RNA, rRNA, tRNA, and snoRNA) were identified and masked using the sequence comparison program Cross_Match [23]. Very few genus-specific and no species-specific chloroplast sequences were available. Vector trimming excised the longest non-masked sequence and further trimming removed low quality bases (less than phred score 20) at both ends of a read. Sequences were discarded if they lacked a polyA tail (to eliminate chloroplast genome encoded sequences), had greater than 5% ambiguous bases, or had fewer than 100 high quality bases (minimum phred score of 20). PolyA tails were searched for by finding the first run of at least 9 A's after the first 350 bases of the sequence.

### Assembly of high quality sequences and annotation

The filtered library file was assembled using the contig assembly program CAP3 [24]. More stringent parameters (-p 90. -d 60) were used to prevent over assembly and help identify potential paralogs. The unigene data set was derived by combining the contig and singleton data sets. Annotation of the unigene data set consisted of pairwise comparison of both the filtered library and the contig consensus library file against the GenBank nr protein database using the fastx3.4 algorithm [25]. Sequences were considered similar only if the expect value cut-off was less than 1e-6. The sequences were also characterized by comparison with the Arabidopsis proteins from TAIR [26], and the Swiss-Prot protein database [27]. Contigs with putative identities were classified into 14 functional groups and then into subgroups within each of these basic groups using a scheme described previously for blueberry [9]. Classification was based on known function of proteins by reference to the BioCyc-MetaCyc: Encyclopedia of Metabolic Pathways website [28], by reference to the gene ontology (Go) database [29], and by searching related abstracts in PubMed [30].

### Primer design and testing

Simple Sequence Repeats (SSRs) were identified in the unigene data set using the CUGISSR.pl script, based on the software SSRIT [31,32] and further filtered for optimal primer development (40–60% GC content and at least 20 base pairs of sequence either side of the motif). Primers were designed from the surrounding sequences using Primer 3 [33]. Primer sequences were designed from individual sequences rather than contigs to avoid non-amplification problems that could result from incorrect sequence assembly. A subset of 33 primer pairs were tested on thornless 'Arapaho' [34], and primocane-fruiting 'APF-12' (Prime-Jim^®^) [1]. DNA extraction, polymerase chain reactions (PCR) and sizing of PCR products were done as described by Stafne et al. [8].

## Authors' contributions

KSL grew the plants and planned the study. KSL and ALD harvested the leaf tissue. ALD extracted the RNA under the direction of LJR. BJC cloned, picked, and checked the library initially for quality. DCH and CAS sequenced the ESTs under the guidance of JPT. MES conducted the trace file processing, sequence annotation, contig assembly, SSR search and EST-SSR primer design under the direction of DSM and JPT. LJR categorized annotated genes into functional categories. KSL supervised the extraction of DNA and testing of EST-SSR primer pairs in PCR. KSL drafted the manuscript and all authors read and approved the final manuscript.

## Supplementary Material

Additional file 1 **Blackberry (*Rubus *L.) EST contigs containing more than five sequences**. Description and accession number of the Entrez protein database sequence that the blackberry sequence most closely matched (match sequence), and functional category of the match sequence. Click here for file

Additional file 2 **Test of SSR primer pairs derived from blackberry (*Rubus *L.) SSR containing EST sequences**. Included are contigs to which the sequences were assembled, sequence similarity matches, repeat motif within the sequence, number of repeats of the motif, placement in an open reading frame (ORF, "Yes" if the SSR was present in the ORF, and "No" if the SSR was not present in the ORF), amplification success, and type of polymorphism detected ("No" for no polymorphism, "Size" for a size polymorphism between the two genotypes tested, or "+/-" for a primer pair that amplified at least one product from one of the two genotypes but not the other). Click here for file
